# Supplementary material for: Drought as a possible contributor to the Visigothic Kingdom crisis and Islamic expansion in the Iberian Peninsula
Source: Nat Commun. 2023 Sep 15;14:5733. doi: 10.1038/s41467-023-41367-7 (PMC10504262; doi:10.1038/s41467-023-41367-7)
Supplement: Supplementary file 1 — Supplementary Information [file 41467_2023_41367_MOESM1_ESM.docx]

**SUPPLEMENTARY INFORMATION**

**Drought as a possible contributor to the Visigothic Kingdom crisis and Islamic expansion in the Iberian Peninsula**

Jon Camuera^1*^, Francisco J. Jiménez-Espejo^1*^, José Soto-Chica^2^, Gonzalo Jiménez-Moreno^3^, Antonio García-Alix^3^, María J. Ramos-Román^4^, Leena Ruha^5,6^, Manuel Castro-Priego^7^

^1^ Andalusian Earth Sciences Institute (IACT), Spanish National Research Council - University of Granada (CSIC-UGR), Granada, Spain

^2^ Department of Medieval History and Historiographic Sciences and Techniques, University of Granada, Granada, Spain

^3^ Department of Stratigraphy and Paleontology, Faculty of Science, University of Granada, Granada, Spain

^4^ Faculty of Education, Mid-Atlantic University, Madrid, Spain

^5^ Natural Resources Institute Finland, Oulu, Finland

^6^ Research Unit of Mathematical Sciences, University of Oulu, Oulu, Finland

^7^ Unit of Archaeology, Department of History and Philosophy, University of Alcalá, Alcalá de Henares, Spain

^*^ Corresponding authors: Jon Camuera and Francisco J. Jiménez-Espejo

E-mail addresses: Jcamuera@gmail.com, Francisco.jimenez@csic.es

**SUPPLEMENTARY INFORMATION**

This Supplementary Information file includes:

- Supplementary Figures 1, 2 and 3
- Supplementary References

**SUPPLEMENTARY FIGURES**


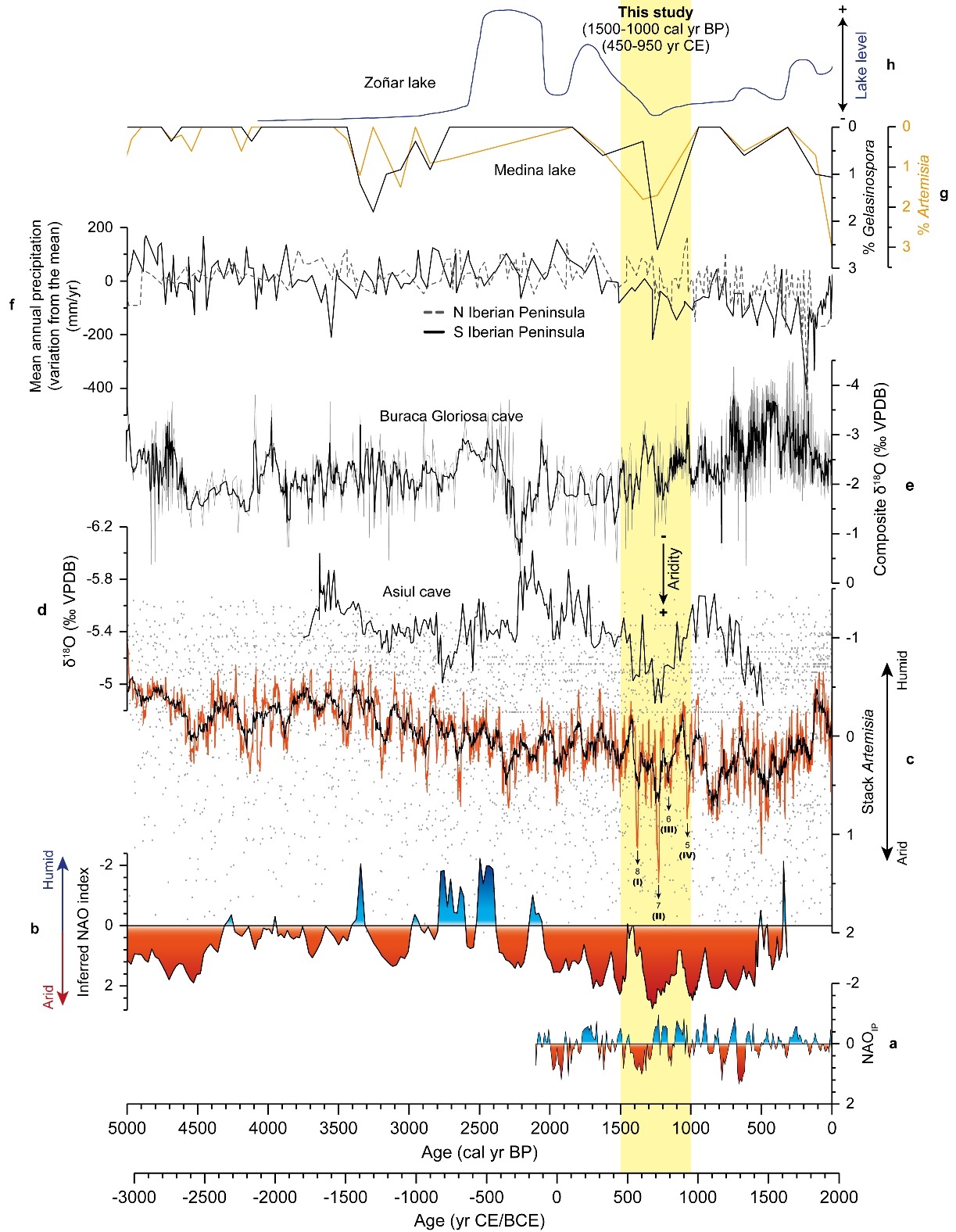


**Supplementary Figure 1 | Comparison between Mediterranean proxy records for the last 5000 years. a,** Reconstructed NAO index for the Iberian Peninsula (inverted axis)^1^. **b,** Inferred North Atlantic NAO index (inverted axis)^2^. **c,** Stack of *Artemisia* from this study (inverted axis), with the black line representing the 45-point moving average and the orange line the 15-point moving average. **d,** δ^18^O record (‰ VPDB, inverted axis) from Asiul cave (N Spain)^3^. **e,** Composite δ^18^O record (‰ VPDB, inverted axis) from Buraca Gloriosa cave (Portugal)^4^. The grey line shows the raw data and the black line the 3-point moving average. **f,** Quantitative mean annual precipitation reconstruction (variation from the mean, in mm/yr) for northern and southern Iberian Peninsula^5^. **g,** Abundance of *Artemisia* and *Gelasinospora* (%, inverted axes) from Medina lake (S Spain)^6^. **h,** Lake level reconstruction from Zoñar lake (S Spain)^7^. The yellow vertical shade shows the period of interest for this study (450-950 CE, 1500-1000 cal yr BP).


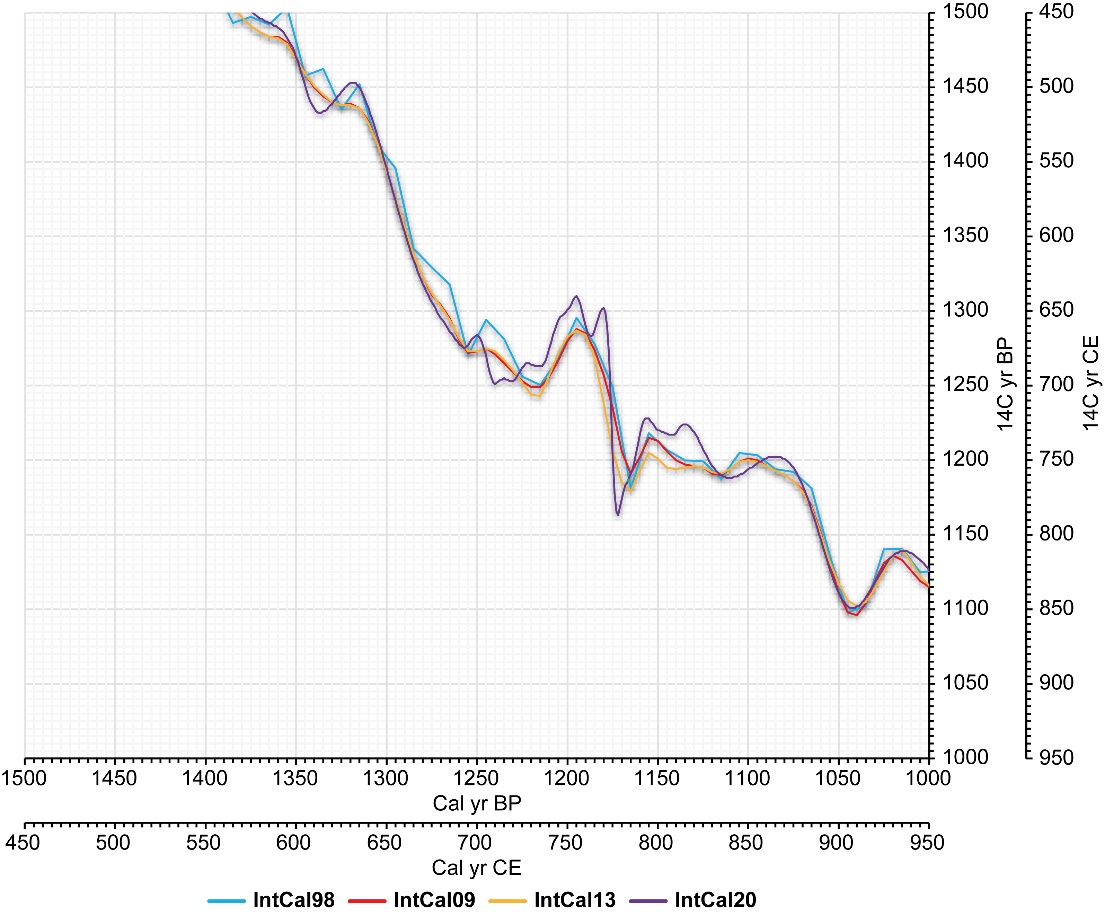


**Supplementary Figure 2 | Comparison between the ^14^C ages and the calibrated ages according to different calibration curves (IntCal98, IntCal09, IntCal13 and IntCal20) for the period of interest (450-950 CE, 1500-1000 cal yr BP).** Since the IntCal04 and the IntCal09 calibration curves for the last 12,000 years are the same^8^, the oldest IntCal04 curve was not included in the figure.


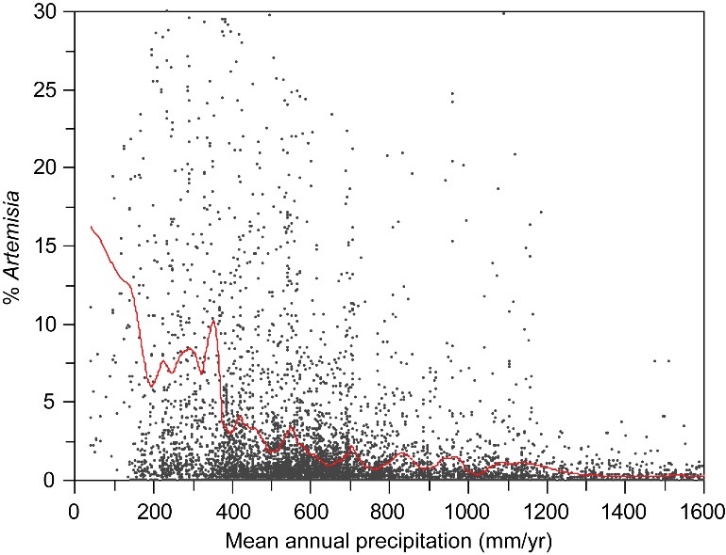


**Supplementary Figure 3 | Recent mean abundance of *Artemisia* (in percentages) with respect to the recent mean annual precipitation according to the EMPDv2 (8217 modern pollen sites) and WorldClimv2.1 data.** The black dots indicate each sample from the EMPDv2 and the red line the smoothing applied to observe the evolution of the abundance of *Artemisia* with the increasing/decreasing precipitation.

**SUPPLEMENTARY REFERENCES**

1 Hernández, A. *et al.* A 2,000-year Bayesian NAO reconstruction from the Iberian Peninsula. *Scientific Reports* **10**, 14961 (2020).

2 Olsen, J., Anderson, N. J. & Knudsen, M. F. Variability of the North Atlantic Oscillation over the past 5,200 years. *Nature Geoscience* **5**, 808-812 (2012).

3 Smith, A. C. *et al.* North Atlantic forcing of moisture delivery to Europe throughout the Holocene. *Scientific Reports* **6**, 24745 (2016).

4 Thatcher, D. L. *et al.* Hydroclimate variability from western Iberia (Portugal) during the Holocene: Insights from a composite stalagmite isotope record. *The Holocene* **30**, 966-981 (2020).

5 Ilvonen, L. *et al.* Spatial and temporal patterns of Holocene precipitation change in the Iberian Peninsula. *Boreas* **51**, 776-792 (2022).

6 Schröder, T., López-Sáez, J. A., van’t Hoff, J. & Reicherter, K. Unravelling the Holocene environmental history of south-western Iberia through a palynological study of Lake Medina sediments. *The Holocene* **30**, 13-22 (2020).

7 Martín-Puertas, C. *et al.* Arid and humid phases in southern Spain during the last 4000 years: the Zoñar Lake record, Córdoba. *The Holocene* **18**, 907-921 (2008).

8 Reimer, P. J. *et al.* IntCal09 and Marine09 Radiocarbon Age Calibration Curves, 0–50,000 Years cal BP. *Radiocarbon* **51**, 1111-1150 (2009).
