# Supplementary material for: Drought as a possible contributor to the Visigothic Kingdom crisis and Islamic expansion in the Iberian Peninsula
Source: Nat Commun. 2023 Sep 15;14:5733. doi: 10.1038/s41467-023-41367-7 (PMC10504262; doi:10.1038/s41467-023-41367-7)
Supplement: Supplementary file 3 — Description of Additional Supplementary Files [file 41467_2023_41367_MOESM3_ESM.pdf]

DESCRIPTION OF ADDITIONAL SUPPLEMENTARY FILES

SUPPLEMENTARY DATA 1 AND 2

The Supplementary Data 1 and Supplementary Data 2 include:

- Supplementary Data 1: The detailed information of the 107 fossil pollen records from Iberia and Morocco used in this study. The table includes *Site nº (map), Sitename, Database, Site ID, Dataset ID, Latitude, Longitude, Altitude, Chronology source, Dating (^{14}C / ^{210}Pb / ^{137}Cs ages ± error) and depths (cm), Age model details* and *References*.
- Supplementary Data 2: The normalized stacks of *Artemisia* and xerophytes based on 100 (for *Artemisia*) and 107 (for Xerophytes) fossil pollen records, together with 3-point, 15-point and 45-point moving averages (also available at www.pangaea.de).
