# Supplementary material for: Drought as a possible contributor to the Visigothic Kingdom crisis and Islamic expansion in the Iberian Peninsula
Source: Nat Commun. 2023 Sep 15;14:5733. doi: 10.1038/s41467-023-41367-7 (PMC10504262; doi:10.1038/s41467-023-41367-7)
Supplement: Supplementary file 6 — Reporting Summary [file 41467_2023_41367_MOESM6_ESM.pdf]

nature portfolio

Corresponding author(s): Jon Camuera and Francisco J. Jiménez-Espejo

Last updated by author(s): Aug 30, 2023

Reporting Summary

Nature Portfolio wishes to improve the reproducibility of the work that we publish. This form provides structure for consistency and transparency in reporting. For further information on Nature Portfolio policies, see our Editorial Policies and the Editorial Policy Checklist.

Statistics

For all statistical analyses, confirm that the following items are present in the figure legend, table legend, main text, or Methods section.

n/a	Confirmed	
☐	☒	The exact sample size (*n*) for each experimental group/condition, given as a discrete number and unit of measurement
☐	☒	A statement on whether measurements were taken from distinct samples or whether the same sample was measured repeatedly
☐	☒	The statistical test(s) used AND whether they are one- or two-sided<br>*Only common tests should be described solely by name; describe more complex techniques in the Methods section.*
☐	☒	A description of all covariates tested
☒	☐	A description of any assumptions or corrections, such as tests of normality and adjustment for multiple comparisons
☐	☒	A full description of the statistical parameters including central tendency (e.g. means) or other basic estimates (e.g. regression coefficient) AND variation (e.g. standard deviation) or associated estimates of uncertainty (e.g. confidence intervals)
☒	☐	For null hypothesis testing, the test statistic (e.g. *F*, *t*, *r*) with confidence intervals, effect sizes, degrees of freedom and *P* value noted<br>*Give P values as exact values whenever suitable.*
☒	☐	For Bayesian analysis, information on the choice of priors and Markov chain Monte Carlo settings
☒	☐	For hierarchical and complex designs, identification of the appropriate level for tests and full reporting of outcomes
☒	☐	Estimates of effect sizes (e.g. Cohen's *d*, Pearson's *r*), indicating how they were calculated

Our web collection on statistics for biologists contains articles on many of the points above.

Software and code

Policy information about availability of computer code

Data collection	The data collection was obtained from the Neotoma database (www.neotomadb.org), the European Pollen Database (www.europeanpollendatabase.net) and privately contacting with authors for fossil records not included in open databases. No software was used for data acquisition.
Data analysis	The data was analyzed using the RStudio version 2022.02.0 (www.rstudio.com).

For manuscripts utilizing custom algorithms or software that are central to the research but not yet described in published literature, software must be made available to editors and reviewers. We strongly encourage code deposition in a community repository (e.g. GitHub). See the Nature Portfolio guidelines for submitting code & software for further information.

Data

Policy information about availability of data

All manuscripts must include a data availability statement. This statement should provide the following information, where applicable:

- Accession codes, unique identifiers, or web links for publicly available datasets
- A description of any restrictions on data availability
- For clinical datasets or third party data, please ensure that the statement adheres to our policy

The fossil pollen data were collected from the Neotoma database (www.neotomadb.org), the European Pollen Database (www.europeanpollendatabase.net) and

privately contacting with authors for the fossil data not included in open databases. For calibration, the Eurasian Modern Pollen Database (Davis et al., 2020) and the WorldClim v2.1 data (www.worldclim.org) was used.

Research involving human participants, their data, or biological material

Policy information about studies with human participants or human data. See also policy information about sex, gender (identity/presentation), and sexual orientation and race, ethnicity and racism.

Reporting on sex and gender	This study does not involve human research participants.
Reporting on race, ethnicity, or other socially relevant groupings	This study does not involve human research participants.
Population characteristics	This study does not involve human research participants.
Recruitment	This study does not involve human research participants.
Ethics oversight	This study does not involve human research participants.

Note that full information on the approval of the study protocol must also be provided in the manuscript.

Field-specific reporting

Please select the one below that is the best fit for your research. If you are not sure, read the appropriate sections before making your selection.

☐ Life sciences ☐ Behavioural & social sciences ☒ Ecological, evolutionary & environmental sciences

For a reference copy of the document with all sections, see nature.com/documents/nr-reporting-summary-flat.pdf

Ecological, evolutionary & environmental sciences study design

All studies must disclose on these points even when the disclosure is negative.

Study description	This study focuses on pollen-based drought events and their influence in the Visigothic Kingdom crisis and the Islamic expansion in the Iberian Peninsula between the 5th - 10th centuries CE. Drought events were obtained using arid-adapted pollen taxa from Iberia and Morocco. The most representative arid-adapted vegetation taxon in the Mediterranean region (i.e., Artemisia) was analyzed under the recently developed Scale-normalized Significant Zero crossing (SnSiZer) analysis. The statistical analysis identified four main strong drought events (at 545-570, 695-725, 755-770 and 900-935 CE) that matched with historical sources and archaeological data, suggesting that these persistent droughts could have contributed to the Visigothic Kingdom crisis and the Islamic expansion in Iberia.
Research sample	The pollen data were obtained from the Neotoma database (www.neotomadb.org), the European Pollen Database (www.europeanpollendatabase.net) and privately contacting with authors for the fossil records not included in open databases. With the combination of both databases, we obtained all the freely available fossil pollen records from Iberia and Morocco, and therefore, a large number of data that allowed a detailed paleoclimate interpretation of the Iberian Peninsula for the period of interest (450-950 CE, 1500-1000 cal yr BP).
Sampling strategy	We selected all the continental fossil pollen records from Spain, Portugal, Andorra and Morocco containing data during the last 5000 years, which resulted in 107 pollen records (Fig. 1).
Data collection	J.C. downloaded the open-source raw pollen data from Neotoma and the European Pollen Database, collecting the pollen records presenting samples during the last 5000 years. We first harmonized the vegetation taxa of the 107 fossil records and obtained the percentages for each taxon and record. We focused on Artemisia data because it is one of the most representative vegetation taxa of the Mediterranean region related with arid conditions. The calibration of the Artemisia pollen abundance with respect to the recent precipitation values was done using the open-source Eurasian Modern Pollen Database and the WorldClimv2.1 data (www.worldclim.org).
Timing and spatial scale	This study collected the fossil pollen records containing data during the last 5000 years. The stacked Artemisia record of the last 5000 years based on the collected 107 fossil pollen records presents 3977 samples. The age chronologies of the fossil pollen records were obtained from the databases, including the chronologies from the original studies and the chronologies produced by the Mapping and Data Accuracy working group (MADCAP) of the European Pollen Database.
Data exclusions	No data were excluded from the analyses.
Reproducibility	This study used the open-source fossil pollen data from Neotoma and the European Pollen Database. Therefore, this work can be reproduced using the freely available data and code. L.R. developed the new SnSizer extension and could freely provide the modified code of the original SiZer to perform the statistical analysis.
Randomization	The pollen records from Iberia and Morocco were randomly and objectively selected according to the age chronologies and location (Iberia and Morocco). Therefore, no randomization was necessary.

Blinding	Blinding is not relevant for this study. The data were acquired from the pollen databases. Given the nature of the data collection (directly from the open-source databases or contacting the authors of the original studies), blinding was not necessary in this study.

Did the study involve field work? ☐ Yes ☒ No

Reporting for specific materials, systems and methods

We require information from authors about some types of materials, experimental systems and methods used in many studies. Here, indicate whether each material, system or method listed is relevant to your study. If you are not sure if a list item applies to your research, read the appropriate section before selecting a response.

Materials & experimental systems

n/a	Involved in the study
☒	☐ Antibodies
☒	☐ Eukaryotic cell lines
☒	☐ Palaeontology and archaeology
☒	☐ Animals and other organisms
☒	☐ Clinical data
☒	☐ Dual use research of concern
☒	☐ Plants

Methods

n/a	Involved in the study
☒	☐ ChIP-seq
☒	☐ Flow cytometry
☒	☐ MRI-based neuroimaging
